# It’s a Family Affair: A Case for Consistency in Family Foundation Giving and Family Firm Community CSR Activity

**DOI:** 10.1007/s10551-023-05424-x

**Published:** 2023-06-03

**Authors:** Cristina Cruz, Hana Milanov, Judit Klein

**Affiliations:** 1 Entrepreneurship and Family Businesses, IE Business School, C/Alvarez de Baena 4, ES 28006 Madrid, Spain; 2 TUM School of Management, Technical University of Munich, Arcisstr. 21, 80333 Munich, Germany

**Keywords:** Family firms, Family foundations, Community CSR, Socioemotional wealth, Instrumental stakeholder theory, Cue consistency, Business ethics

## Abstract

Although most business-owning families (BOFs) that operate large family firms practice community social engagement both in private via family foundations and in the business domain via community corporate social responsibility (CSR) programs, the relationship between their activities in the two domains remains unclear. Prior literature speculates that BOFs will deprioritize firms’ community CSR when they have family foundations as more efficient vehicles to achieve socioemotional wealth (SEW), which would imply that such BOFs are less ethical in operating their firms. We contrast these speculations by enriching the socioemotional wealth (SEW) approach with instrumental stakeholder theory and cue consistency arguments and theorize that BOFs seek to ensure consistency between their activities in the two domains. Using data from 2008 to 2018 on the 95 largest US public family firms whose BOFs also operate private foundations, we show a positive relationship between family foundation giving and firm community CSR activity. Furthermore, we provide evidence for the boundary conditions of this relationship, showing that it is weaker for firms that do not share the family’s name and stronger for those firms with family leaders who also lead their families’ foundations.

## Introduction

Business-owning families (BOFs), defined as collections of individuals related by consanguinity or marriage who together control the strategy of at least one firm (Nason et al., 2019), commonly endorse and invest in community social activities (Lumpkin & Bacq, 2022). Their community social engagement involves “giving back to society,” but it also has an instrumental purpose for BOFs since it helps them preserve and enhance their socioemotional wealth (SEW) (Cennamo et al., 2012)—i.e., the affective endowment a family derives from its ownership of a firm (Gómez-Mejía et al., 2007). By pursuing their communities’ wellbeing, BOFs develop social capital, an important source of SEW (Lumpkin & Bacq, 2022), and enhance their images and reputations, another key SEW dimension (Berrone et al., 2012).

Scholars utilizing SEW theorizing to examine BOFs’ community social engagement have focused exclusively on the actions they take via their family firms, by examining firm community CSR activity (Cruz et al., 2014). This focus overlooks the fact that BOFs also socially engage with their communities via private channels (Feliu & Botero, 2016; Gersick, 2006; Lungeanu & Ward, 2012; Moody et al., 2011; Van Gils et al., 2014), where family foundations are the most common way of doing so (Feliu & Botero, 2016).Indeed, 47% of the BOFs that operate the world’s largest family firms have a family foundation (Richards et al., 2016), and family foundation giving has almost tripled since the early 2000s, climbing from US$12.4 billion in 2002 to US$34.6 billion in 2018 (Giving USA 2019).

The prevalence and economic importance of family foundations raises the questions of *if* and *how* this form of private domain community social engagement might influence BOFs’ community social engagement in the business domain, and specifically their community CSR. A few studies speculate that when BOFs pursue high levels of community social engagement via family foundation giving, they selectively lower their firms’ community CSR activity (Atkinson & Galaskiewicz, 1988; Block & Wagner, 2014). These studies assume that family foundations are more efficient avenues for BOF accruement of the SEW benefits associated with community social engagement than activities mediated through their family firms (Block & Wagner, 2014). This speculation implies that family foundation giving effectively makes BOFs less ethical in how they operate their firms, by reducing their community CSR activities. Thus an empirical investigation of this speculative relationship between private family foundation giving and firm community CSR activity is relevant for business ethics research.

In this study, we argue that using a SEW lens alone offers a limited view of BOFs’ community social engagement approaches and counter this extant speculation about a substitution effect between family foundation giving and firm community CSR activity. Under SEW logic, BOFs will choose the most efficient vehicle for community social engagement (either their firms or the foundations) that preserves and enhances their SEW. However, this reasoning ignores the importance of the perceived authenticity of BOFs’ community social engagement among stakeholders—of ensuring that BOFs meet “the true, underlying purpose of the actions stakeholders expect” (Cuypers et al., 2016, p. 176). Meeting this perceived authenticity standard is a potent challenge for BOFs that operate large family firms, as they are constantly pressured to justify “their status and conspicuous privileges” (Gray & Kish-Gephart, 2013, p. 678).

We enrich SEW theorizing through the application of cue consistency theory (Anderson, 1981) within the broader framework of the instrumental stakeholder approach (Jones, 1995) to argue that BOFs only gain community stakeholder support (and its attendant SEW benefits) when stakeholders perceive their community social engagement efforts as authentic. Cue consistency theory suggests that if BOFs selectively use family foundations as a more efficient way to gain SEW, and reduce their community CSR engagement (as prior literature suggests; see Block & Wagner, 2014), stakeholders may detect the inconsistency in their activities between these domains, and thus perceive family foundation’s activities as inauthentic. This may not only counteract family foundation giving’s apparently superior efficacy as a means of accruing SEW benefits but also, given the strong identity connection between BOFs and their associated entities (Berrone et al., 2010), it may damage BOFs’ reputation and thus precipitate a SEW loss. Hence, we hypothesize a positive relationship between family foundation giving and firm community CSR activity. Additionally, we suggest that the ease with which stakeholders can connect a BOF’s community social engagement across the private and business domains acts as an important boundary condition. Specifically, we refine our model by theorizing and empirically validating two boundary conditions—(1) name congruence (i.e., whether a family foundation and family firm have the same name), and (2) leadership congruence (i.e., whether specific members of a BOF have leadership roles in both a family foundation and family firm)—that influence the extent to which a BOF seeks to maintain a complementary relationship between its levels of community social engagement across these two domains.

To test our hypotheses, we use a comprehensive dataset of 95 US BOFs that own both a family foundation and a family firm listed in the Fortune 1000 rankings. We examine BOFs’ community social engagement via their family foundations giving and firm community CSR activity between 2008 and 2018. This examination supports our hypotheses.

Our research contributes to the literature in several ways. First, we employ cue consistency theorizing to extend extant SEW research. Theorizing through SEW logic alone leads to the expectation that BOFs will selectively pursue the most effective means to accruing SEW benefits, with ethical implications for lowering community CSR when BOFs have active foundations. Complementing SEW with cue consistency prompts consideration of *why* and *when* they might engage with particular stakeholder groups holistically: in order to avoid penalties stemming from perceptions of inauthenticity, especially relevant when stakeholders can easily connect the BOF with associated entities (in the case of name and leadership congruence). Hence, our enrichment of SEW theorizing with cue consistency theorizing contributes to the literatures on family business and ethics by challenging depictions of BOFs as entities that generally prefer a selective approach in their social engagement with stakeholders (Cruz et al., 2014; Zientara, 2015). To this end, while our study supports the notion that SEW concerns foster ethical behaviors among BOFs (Astrachan et al., 2020; Reck et al., 2022), we broaden this concept by theorizing that SEW preservation can act as a prosocial stimulus that drives BOFs’social engagement not only *through* their family firms but also *beyond* the firm’s boundaries.

Second, we contribute to the conversation in business ethics studies on authenticity in CSR activities. We validate the relevance of the cue consistency mechanism in the CSR literature, by moving beyond the established importance of ensuring consistency across CSR activities *within* the business domain (Cuypers et al., 2016; Scheidler et al., 2019) and demonstrating the importance of ensuring consistency *across* the business and private domains for BOFs seeking to accrue SEW benefits with community stakeholders.

Finally, we provide evidence of boundary conditions on efforts to achieve authenticity by showing that BOFs’ prioritization of consistency in levels of community social engagement across the business and private domains varies (Zellweger et al., 2013) depending on the extent to which stakeholders struggle to distinguish between the actions of the family and the firm (Deephouse & Jaskiewicz, 2013). This contributes to the growing interest in explaining the heterogeneity of family firms’ social behaviors (Labelle et al., 2015; Marques et al., 2014; Van Gils et al., 2014) and, more broadly, the drivers behind their ethical practices.

### SEW, Instrumental Stakeholder Theory, and BOF Community Social Engagement

The behavioral agency model exploring how BOFs behave in the pursuit of SEW has increasingly become the theoretical foundation for most family business research on CSR issues (Bingham et al., 2011; Cruz et al., 2014; Labelle et al., 2015). From a SEW perspective, BOFs decide to socially engage with their communities in ways that align with their motivations to accrue and preserve SEW (Cennamo et al., 2012). The “binding social ties” dimension of SEW assumes that when BOFs establish strong social ties with their communities, they develop social capital that accrues as SEW, which they then strive to preserve and transfer across generations (Berrone et al., 2012). Moreover, because of the strong link between a family’s identity and that of the firms it owns (Berrone et al., 2010; Deephouse & Jaskiewicz, 2013; Dyer & Whetten, 2006), socially engaging with their communities through family firms enhances the family’s reputation, another key SEW dimension (Berrone et al., 2012).

Beyond socially engaging with their communities via a family firm (in the form of community CSR activity), BOFs may pursue SEW rewards via a family foundation, as such an entity “transforms private wealth into a tax-favored and socially credible institution” (Ylvisaker, 1990, p. 333). Family foundations—independent entities established by individual donors and additional family members for the purposes of engaging in philanthropic work (Gersick, 2006; Lungeanu & Ward, 2012)—are increasingly relevant forces in US philanthropy. Nevertheless, despite their philanthropic nature (Feliu & Botero, 2016), the growth of family foundations has generated growing public—and community stakeholder—scrutiny (Rey-García & Puig-Raposo, 2013; Sullivan, 2015).^1^ Proponents of “philanthro-shaming” argue that family foundations perpetuate wealthy BOFs’ privileges by yielding them immediate income tax deductions, as well as longer-term tax advantages (for specific tax benefits, see Silk & Lintott, 2002). These benefits are highly visible to external audiences, thanks in part to the transparency rules imposed by the US Congress in response to reported abuses of the family foundation structure for individual and familial enrichment (Silk & Lintott, 2002). As one family philanthropy advisor puts it, “a private foundation is about the least private thing you can have … By meeting Internal Revenue Service reporting requirements, you’re opening up your affairs to the world” (Sullivan, 2019). Hence, operating a family foundation opens a BOF’s community social engagement to unique public scrutiny.

Instrumental stakeholder theory (Jones, 1995) suggests that this scrutiny may influence a BOF’s SEW in so much as the family will only reap the community stakeholder support and its attendant SEW benefits from its community social engagement efforts if key stakeholders view these efforts as authentic (Cuypers et al., 2016), rather than motivated by self-interest (Godfrey, 2005; Scheidler et al., 2019). Authenticity is a particularly sensitive topic for the BOFs that operate the world’s largest family firms, given their characterization as members of an elite social category (Palmer & Barber, 2001),^2^ and the pressures they face to justify the social and economic privileges that come with this elite status (Nason et al., 2019). Public scrutinization of the perceived authenticity of wealthy BOFs’ private giving is often influenced by media reports that direct observers’ attention to the firms that generate these families’ wealth—especially when a BOF’s family foundation engages in high levels of private giving. For example, a *Washington Post* article on Walton family members’ philanthropic activities described them as “the nation’s richest [family] through its ownership stake in Walmart” (Soskis, 2014). Some critics go so far as to accuse BOFs of using private giving to “sweep under the carpet corporate malpractices” (Rhodes & Bloom, 2018). For instance, in 2014 the New York City Council accused the Waltons’ family foundation of using its giving as a “cynical public relations campaign that disguises Walmart’s backwards anti-job agenda” (Covert 2014).

Given BOFs’ visibility, the public scrutiny family foundations engender, and the ease with which stakeholders can connect BOFs to the foundation and the firm they own, BOFs—driven by SEW concerns—are likely to take special care to ensure that key stakeholders perceive their community social engagement to be authentic.

In the next section, we build on cue consistency theory (Slovic, 1966) to theorize about how stakeholders’ abilities to simultaneously observe BOFs’ community engagements in the private and business domains can shape their perceptions of the authenticity of these engagements.

### Cue Consistency Theory and BOFs’ Community Social Engagement

The CSR literature stresses that, because the motives behind BOFs’ social engagements are largely unobservable, stakeholders default to focusing on cues—the observable attributes of social engagement efforts (Wang et al., 2020)—to attempt to gauge the authenticity of these actions. When a BOF’s social engagement involves multiple observable attributes, they must ensure consistency between these distinct informational cues if they want stakeholders to perceive them as authentic and thus ensure stakeholder support (e.g., Scheidler et al., 2019; Wang & Choi, 2013).

These cues can take the form of anything from a firm’s own communication about its community engagement (e.g., via its website and in recruitment materials on job fairs) (Jones et al., 2014) to media reports on a firm’s CSR activities (Wagner et al., 2009), and beyond. In the context of this study, BOFs’ social engagements themselves constitute important cues. Notably: (1) The amount of BOF’s family foundation giving can be an important quantitative cue that helps stakeholders assess how substantively they engage in philanthropic activities; (2) CSR activities are important cues, because these help stakeholders assess the firm’s values (e.g., Brammer et al., 2006).

The literature on the importance of ensuring consistency between these types of cues fittingly draws, sometimes implicitly (Cuypers et al., 2016; Scheidler et al., 2019) and sometimes explicitly (De Roeck et al., 2016; Ghosh, 2018; Rodrigo et al., 2019), on cue consistency theory (Anderson, 1981; Slovic, 1966). Although originally developed in the psychology literature, scholars productively applied it in the management literature on CSR (De Roeck et al., 2016; Ghosh, 2018; Wagner et al., 2009). The theory holds that when observers encounter a set of consistent cues regarding an actor or an issue, they can easily make an overall judgment by simply “adding [them] up,” with the side effect that their confidence in these cues’ apparent indications increases as well (Slovic, 1966). However, when faced with inconsistent cues, observers tend to focus on the more negative among them—a phenomenon the theory refers to as a “negativity bias” (Maheswaran & Chaiken, 1991; Miyazaki et al., 2005).

For stakeholders observing firms’ behaviors across domains, any inconsistency can “hinder their reactions to [positive] cues or even create a negative response if they develop judgments of hypocrisy or feelings of betrayal” (De Roeck et al., 2016, p. 1147). Importantly, negativity bias is more likely to be exhibited in skeptical environments, contexts in which people are especially vigilant about the authenticity behind cues (Maheswaran & Chaiken, 1991; Miyazaki et al., 2005), such as in the observation of CSR practices (Hsueh, 2018). For example, encountering inconsistencies between how a firm publicly communicates about its CSR and how the media portrays it seems to lead individuals to exhibit a negativity bias, which leads them to perceive these CSR practices as inauthentic and to hold negative attitudes towards what they perceive to be hypocritical firms (Wagner et al., 2009).

In our context, heightened public skepticism of and scrutiny regarding family foundations means stakeholders are likely to notice inconsistencies in BOFs’ community social engagement across domains, especially if these involve high levels of private family foundation giving and comparatively low levels of business-domain community CSR. Cue consistency studies suggest such a disparity would likely trigger negativity biases (Miyazaki et al., 2005). Accordingly, a BOF lowering its firm’s community CSR activities as it increases family foundation giving would likely prompt a negativity bias among stakeholders observing that inconsistency, as the negative cue (lower firm community CSR activity) will outweigh the positive cue (higher foundation giving) in assessments of the overall authenticity of the family’s community social engagement. In this case, community stakeholders will likely perceive an ulterior motive behind the BOF’s social engagements. This dynamic would invalidate the benefits for a BOF of selectively favoring family foundation giving as a more efficient means of accruing SEW via social capital (c.f. Block & Wagner, 2014), as stakeholder perceptions of their apparent hypocrisy would hinder the family in their efforts to build enduring relationships with their communities. Moreover, the strong link between BOF’s identity and that of the entities it owns (Dyer & Whetten, 2006) means that negative judgments of this observed incongruity could backfire, damaging the BOF’s image and reputation and thus generating a potential loss of SEW.

Hence, to maximize potential SEW gains in the form of community stakeholder support while minimizing potential SEW losses from negative reputational spillovers between foundation, firm, and family, we argue that BOFs will attempt to match high levels of private community social engagement via family foundation giving with correspondingly high social engagement in the business domain via community CSR activity. This leads us to hypothesize:

#### Hypothesis 1 (H1):

BOFs’ family foundation giving is positively related to their family firms’ community CSR activity.

### Name Congruence, Family Foundation Giving, and Community CSR

The literature on CSR in the family business context commonly assumes that connections between a BOF and its firm are easily observable to stakeholders (Berrone et al., 2010; Zellweger et al., 2013). However, in reality, the visibility of these connections varies. For example, stakeholders might relatively easily relate the Kellogg family and/or the Kellogg Family Foundation to the Kellogg Company, but may not immediately connect the Dorrance family and/or the Dorrance Family Foundation with the Campbell Soup Company. This type of name (in)congruence—i.e., whether a family’s name appears in a firm’s name—is not only an important symbol of family members’ identification with a firm (Dyer & Whetten, 2006; Zellweger et al., 2013), and it is also closely associated with behavioral consequences (Deephouse & Jaskiewicz, 2013; Rousseau et al., 2018) as it represents an externally observable indicator of the link between a family and a firm.

In our context, which involves cues derived from two distinct domains, we argue that the relative ease (or difficulty) with which community stakeholders can observe connections between a BOF’s family foundation and firm—and thus associated cues linked to one entity with those linked to the other—will moderate cue consistency effects. The institutionalized connection between family and firm, in the form of the congruence of their names, both increases the family’s visibility in communities where the firm operates (Zellweger et al., 2013), and also helps stakeholders more easily connect—and notice any inconsistencies between—the actions of the family and those of this associated entity (Block & Wagner, 2014). Research shows that, in the absence of name congruence, family members are less worried about the reputation of their families’ firms, given the greater difficulty observers face in associating the two (Deephouse & Jaskiewicz, 2013; Rousseau et al., 2018).

The frequent overlap between BOF and family foundation names speaks to the role of family foundation giving as a relevant, easily observable cue for a BOF’s private domain community social engagement.^3^ In “A Guide to Naming Your Foundation,” Foundation Source (2020) notably states: “Like choosing a name for your child, the act of choosing a name for your foundation forces you to think about your intentions and the impression you want to create.” This suggests that, by including a BOF’s name within its foundation’s name, a BOF also specifically intends to ensure that the foundation will act as a clear and visible representation of the family’s public image as a social benefactor—adding to its validity as an important cue of private social community engagement.

Although legacy motives lead most BOFs to name their foundation after their family, often as a tribute to their firm’s founder (Rey-García & Puig-Raposo, 2010) or another specific family member, not all family firms carry the BOF’s name. Hence, community stakeholders will likely have a harder time connecting cues between a family’s private and business-domain community social engagement in the case of BOFs whose firms’ names do not include their —and thus often their family foundation’s—name (e.g., the Dorrance family and the Dorrance Family Foundation versus the Campbell Soup firm). Thus there is likely less of a risk that they will incur a negativity bias if BOFs’ engagements in these distinct domains diverge from each other. This may, then, weaken the importance of cue consistency for a BOF and its associated entities, and correspondingly weaken the positive relationship between a BOF’s family foundation giving and its firm’s community CSR activity. Formally:

#### Hypothesis 2 (H2):

The positive association between BOFs’ family foundation giving and their family firms’ community CSR activity is less positive for family firms that do not share the BOFs’ name.

### Leadership Congruence, Family Foundation Giving, and Community CSR

Evidence from large family firms suggests that family members involved in managing a BOF’s philanthropy also tend to be active in their businesses, often as a firm CEO (in 40% of cases) or as a board member (in another 40% of cases) (Richards et al., 2016).^4^ For example, members of the Sulzberger family, who own and operate the *New York Times*, hold leadership positions in both their firms and their family foundations. Since CEO and chair positions are arguably the strongest personifications of an organization (Filkenstein et al., 2009), they attract significant public attention. Indeed, the CEOs and chairs of the largest firms in the US often achieve near- “celebrity status,” as evidenced by extensive media coverage of these individuals (Hayward et al., 2004; Love et al., 2017). This is consequential, because they are responsible for the firm’s actions (Carpenter et al., 2004) and are commonly the most salient targets for any popular blame for these actions (Gomulya & Boeker, 2016).

Cue consistency theory suggests that when the same family members personify both a family’s private domain foundation and business-domain community CSR, ensuring consistency in engagement across these domains will likely be more important to that BOF. In such a context of leadership congruence, stakeholders can relatively easily connect cues related to distinct private and business entities. This context also makes it easier for stakeholders to assign responsibility for social engagement in both domains to the same family leaders, potentially magnifying any negativity biases that may result from inconsistent engagement across them through personalization of responsibility. Formally, this leads us to hypothesize:

#### Hypothesis 3 (H3):

The positive association between BOFs’ family foundation giving and their family firms’ community CSR activity is more positive for family firms that share family leadership positions with the BOFs’ family foundation.

## Data and Methodology

### Sample and Data Collection

Our sample includes 95 US family firms, observed from 2008 until 2018, that represent a broad cross-section of industries and are all operated by BOFs that also run their own private family foundations. This amounts to 773 observations.^5^ We identified our sample by checking the Fortune 1000 rankings against the Compustat Global and Bloomberg databases’ information on ranked firms’ ages, finances, sectors, and sizes. We excluded firms for which we could not find financial information, as well as firms in the financial, government, and utilities sectors, following the example of prior studies on listed (Fama & French, 1992) and family firms (Villalonga & Amit, 2006), in order to avoid potential distortions when comparing firms’ financial information. We then manually inspected each remaining ranked firm’s proxy statement and website to identify which among them were family firms, and the BOF behind each of them. We categorized companies as family firms if an individual or family owned a minimum 5% of its shares, and at least one family member sat on their boards of directors (Cruz et al., 2014; Villalonga & Amit, 2006). When we identified several families involved in a company’s ownership and/or management, we applied the “ultimate owner” criterion, identifying a focal family based on which owned the highest percentage of shares in the company (Cruz et al., 2014; Miller et al., 2011; Villalonga & Amit, 2006).

Following Cruz et al. (2014), we used the CSRHub database to gather data on our dependent variable: firms’ community CSR activity. We collected family foundation data from the Foundation Directory Online (FDO) database (Lungeanu & Ward, 2012), a knowledge bank managed by the Foundation Center, a leading source of information on global grant-makers. The FDO includes exhaustive information on all approximately 140,000 US foundations, collected from 35 data sources, including but not limited to IRS tax filings (Forms 990 and 990-PF), grant-maker websites, foundations’ annual reports, printed application guidelines, and popular press reports. We primarily consulted the IRS tax filings archive, which contains information about foundations’ contributors and governing bodies—for example, the names of their directors, trustees, and managers. We used our list of identified “ultimate owners” to search the Database for information on the potential existence of family foundations linked to the BOF that operated each family firm in our sample.

Because the United States does not define a family foundation as a distinct legal entity (Moody et al., 2011), we mirrored the methods of previous studies, using name congruence between a family and a foundation as one means of identifying family foundations (Gersick, 2006; Lungeanu & Ward, 2012; Moody et al., 2011). Specifically, we matched the names of foundations with the names of BOFs on our list. We also scrutinized IRS filings from our study period to see whether family members from the firms on our list served on foundation boards, and whether the sources of these foundations’ assets and contributions received were primarily linked to the same BOF (Feliu & Botero, 2016; Gersick, 2006; Lungeanu & Ward, 2012; Moody et al., 2011). We also inspected whether the FDO database categorized a foundation, or the foundation identified itself, as a family foundation (by using the term “family” in its name) (Moody et al., 2011). We included foundations in our sample if they had at least two of the above-mentioned characteristics (Moody et al., 2011). We did not include foundations that had direct connections to family firms (e.g., corporate foundations endowed and managed by a family firm) in order to ensure the separation of the private family and business domains. As detailed below, we introduced a separate control in our models to account for the possible presence of corporate foundations.

### Measures

#### Dependent Variable

Our dependent variable, firm *community CSR activity* reflects a firm’s level of community social engagement, calculated based on CSRHub’s scoring system. The CSRHub database provides social, environmental, community, and governance ratings on around 10,000 companies from 134 industries and 154 countries. According to CSRHub’s documentation, the community CSR category focuses on a company’s level of community social engagement, where its community is broadly conceptualized to include constituents in and around the locations where it operates, as well as locations surrounding its value chain, whether on the supply chain or customer side. Specifically, CSRHub breaks community CSR activity into three sub-categories: (1) a firm’s philanthropic relationship, whether through financial or in-kind giving or staff volunteering, with communities in which it is embedded; (2) a firm’s transparency and commitment to respecting human rights near its proposed or current areas of operation; and (3) a firm’s responsibility for the development, design, and management of its products and services, with respect to their potential impact on customers and their communities. The score is measured in *t* + *1* to ensure that our control variables can affect CSR decisions in a timely manner.

#### Independent Variable

We collected the data on *family foundation giving* (disbursements) from the IRS tax filings of each foundation in our sample (Brody & Strauch, 1990; Lungeanu & Ward, 2012). Our foundation giving variable reflects currently deployed capital, and captures a BOF’s near-term decisions about its foundation’s social impact (Johnson, 2018). Thus, this variable is the closest (monetary) approximation we have access to for a BOF’s private community social engagement. Foundation giving also offers a dynamic measure of this type of BOF’s social engagement. Specifically, although US foundations are obliged by law to disburse 5% of their average net assets to qualified public charities each year (Silk & Lintott, 2002), family foundations also consider long-term horizons in planning their grant-making activities (Feliu & Botero, 2016). Legally speaking, “payouts exceeding the annual minimum requirement may be carried forward five years and applied toward satisfying the minimum payout requirements in those years” (Hayes & Adams, 1990, p. 392). This means that tracking actual foundation giving each year can help us account for any long-term tendencies (including possible dips and spikes) in giving that may affect a family’s decisions about social engagement in the private domain. Since wealthier BOFs can give more than less wealthy BOFs, we scale foundation giving to make it comparable across different families. Given the difficulty of obtaining accurate yearly data on private family wealth, we scaled the variable by dividing it by family firm assets. We did so because family firms are typically the primary source of wealth for BOFs (Anderson et al., 2003), and the public expects the family owners behind larger firms to enjoy more wealth and give accordingly.

#### Moderator Variables

We operationalized *name congruence* as a dummy variable and assigned it a value of 1 if a family name appears in a firm’s name (Dyer & Whetten, 2006; Zellweger et al., 2013). We operationalized *leadership congruence* as a dummy variable and assigned a value of 1 if the family foundation and firm shared key leadership positions (i.e., when the family firm’s CEO and/or chair were on the foundation board of trustees).

#### Control Variables

Guided by the literature on CSR and family businesses, we introduced a number of controls. We controlled for *firm size*, measured as the natural logarithm of the firm’s total assets, because larger companies are more likely to engage in CSR due to closer stakeholder scrutiny (Block & Wagner, 2014; Cruz et al., 2014; Labelle et al., 2015). We also controlled for several financial and market performance variables, because the financial situation of a company can influence its community CSR engagement capabilities. Specifically, we introduced a variable for *return on assets* as a proxy for a firm’s accounting profitability (Block & Wagner, 2014). We introduced a long-term *debt-to-asset ratio* variable, measured as a firm’s financial leverage as the percentage of its assets financed by long-term debt (Block & Wagner, 2014; Cruz et al., 2014). We controlled for firm market growth opportunities, using *Tobin’s Q*, calculated as the sum of the market capitalization ratio and the book value of debt as a percentage of a firm’s total assets (Cruz et al., 2014; Dyer & Whetten, 2006). Finally, we also controlled for *firm age* using its natural logarithm because a firm’s maturity can influence its attitude towards costly investments like CSR (Berrone et al., 2010; Cruz et al., 2014).

We further controlled for family firm-specific variables identified as important in CSR literature (Block & Wagner, 2014; Cruz et al., 2014; Miller et al., 2011). Specifically, we controlled for *family ownership*, measured as the percentage of shares owned by a firm’s focal family; *family management,* coded as 1 when a family member served as the CEO and/or chair of a family business; and *family board control*, operationalized as the ratio of the number of family members involved in the board to the total number of board members (Labelle et al., 2015). We also controlled for *family generation*, assigning a firm the value 1 if a second- or later-generation family member was involved in its operations (Cruz & Nordqvist, 2012).

We wanted to ensure that we ruled out other forms of family firm community social engagement that might affect a given business’s CSR behaviors. Consulting the FDO database, we identified two common means of allocating corporate resources to support social causes beyond CSR: *corporate giving programs* and *corporate foundations* (Feliu & Botero, 2016). Some companies use both of these vehicles for charitable donations. However since corporate foundations are separate, independently governed legal entities, unlike corporate giving programs, (Feliu & Botero, 2016), we controlled for two separate dummy variables, one for each vehicle.

Finally, although controlling for family foundation size would have introduced problems to our analysis given its high correlation with foundation giving, we still wanted to contextualize foundation giving amounts, and to distinguish families that merely obey the 5% minimum rule on annual spending of their foundation’s assets from those exceeding this threshold. Therefore, we calculated *foundation generosity*, measured as a ratio of foundation spending to assets across our sample years and including median spending percentage across years in our models. We added *year* dummy variables to control for any year effects that might have affected corporate spending, and for dynamics in the public markets. We also included *industry dummies* for firms’ three-digit SIC codes (Block & Wagner, 2014), using the construction industry as a reference category.

## Results

Scholars working with panel data must first choose between fixed- and random-effects model specifications. Therefore, we applied the Hausman test (Wooldridge, 2010), which rejected the null hypothesis that the coefficients between fixed and random effects were systematically different (*p* < 0.675), suggesting that random-effects analysis was appropriate. We used robust standard errors in all our estimation models to account for heteroscedasticity. To guard against multicollinearity, we calculated variance inflation factor (VIF) values. Our multicollinearity diagnostics revealed no critical values and had a mean VIF of 5.78, well under 10 (Neter et al., 1996).

We present our descriptive statistics and correlations in Table 1, and our results in Table 2. In H1, we predicted that BOF’s family foundation’s giving would be positively related to their family firm’s community CSR activity. In Table 2, Model 2 shows that the coefficient for family foundation giving is positive (*β* = 1.16, *p* < 0.05), which supports H1.

**Table 1:** Means, standard deviations, and correlations

		Mean	St.Dev	1	2	3	4	5	6	7	8	9	10	11	12	13	14	15
1	Community CSR (t + 1)	49.80	8.33															
2	Firm size (ln)	8.78	1.44	0.05														
3	Return on assets	0.06	0.07	0.22	0.09													
4	Tobin's Q	1.91	1.06	0.19	0.07	0.53												
5	Debt-to-asset ratio	0.24	0.22	– 0.03	0.18	– 0.15	– 0.02											
6	Firm age (ln)	3.41	0.80	0.05	0.02	0.03	0.03	-0.22										
7	Family ownership	0.33	0.27	0.01	– 0.06	– 0.07	– 0.02	0.24	– 0.11									
8	Family management	0.74	0.44	– 0.11	–0.03	-0.08	-0.09	– 0.05	– 0.20	0.22								
9	Family board	0.19	0.11	– 0.08	– 0.19	– 0.02	– 0.11	0.00	0.05	0.31	0.38							
10	Family generation	0.56	0.50	0.03	– 0.15	– 0.04	– 0.08	0.01	0.30	0.12	– 0.10	0.31						
11	Corporate giving program	0.23	0.42	0.05	0.14	0.11	0.08	0.08	– 0.14	0.06	0.16	0.12	0.01					
12	Corporate foundation	0.39	0.49	0.19	0.15	0.07	0.06	– 0.02	0.12	0.04	– 0.20	– 0.11	0.04	– 0.28				
13	Foundation generosity	0.18	0.26	– 0.10	0.11	– 0.03	0.02	0.06	0.11	0.08	– 0.18	– 0.12	– 0.14	– 0.15	0.07			
14	Name congruence	0.27	0.45	–0.03	0.03	– 0.09	– 0.06	0.10	0.15	– 0.03	– 0.07	0.11	0.28	– 0.03	– 0.14	-0.02		
15	Leadership congruence	0.25	0.43	– 0.18	0.00	– 0.12	– 0.07	0.12	− 0.15	0.08	0.34	0.12	0.00	0.10	− 0.07	0.06	− 0.17	
16	Foundation giving	0.18	0.51	0.18	0.03	0.13	0.11	− 0.07	0.07	− 0.02	− 0.06	− 0.06	− 0.04	0.07	− 0.05	0.11	− 0.03	− 0.13

*Correlations above |0.08| are significant at *p* < 0.05

**Table 2:** Results: Random-effects regressions for community CSR (*t* + *1*)

	Model 1		Model 2		Model 3		Model 4		Model 5	
Firm size (ln)	− 0.28	(0.49)	− 0.28	(0.47)	− 0.26	(0.48)	− 0.18	(0.50)	− 0.16	(0.50)
Return on assets	7.81*	(3.72)	7.39*	(3.77)	7.24†	(3.75)	6.94†	(3.77)	6.78†	(3.76)
Tobin's Q	0.20	(0.37)	0.13	(0.39)	0.10	(0.40)	0.12	(0.38)	0.09	(0.38)
Debt-to-asset ratio	0.77	(2.31)	0.95	(2.28)	0.66	(2.27)	1.36	(2.22)	1.05	(2.21)
Firm age (ln)	1.37	(0.95)	1.23	(0.95)	1.17	(0.95)	1.33	(0.98)	1.26	(0.98)
Family ownership	− 2.18	(2.52)	− 2.08	(2.48)	− 2.09	(2.49)	− 1.97	(2.52)	− 1.99	(2.53)
Family management	0.51	(1.00)	0.25	(1.03)	0.27	(1.03)	0.23	(1.02)	0.25	(1.01)
Family board	− 0.97	(5.58)	0.35	(5.79)	0.33	(5.76)	0.52	(5.81)	0.50	(5.77)
Family generation	1.27	(1.34)	1.31	(1.38)	1.25	(1.39)	1.09	(1.40)	1.02	(1.40)
Corporate giving program	1.81	(1.15)	1.90†	(1.14)	1.91†	(1.14)	1.97†	(1.16)	1.98†	(1.16)
Corporate foundation	3.11*	(1.37)	3.20*	(1.36)	3.06*	(1.34)	3.32*	(1.39)	3.18*	(1.36)
Foundation generosity	− 1.22	(3.12)	− 1.39	(3.12)	− 1.85	(3.09)	− 2.25	(3.32)	− 2.76	(3.29)
Name congruence	0.07	(1.48)	0.09	(1.45)	− 0.30	(1.48)	− 0.06	(1.42)	− 0.48	(1.44)
Leadership congruence	− 0.48	(1.50)	− 0.34	(1.50)	− 0.34	(1.51)	− 1.89	(1.37)	− 1.91	(1.38)
Foundation giving			1.16*	(0.52)	0.86*	(0.40)	1.01*	(0.48)	0.70†	(0.38)
Found. giving x name congruence					2.53**	(0.94)			2.73**	(0.94)
Found. giving x leadership congruence							19.67†	(10.48)	19.94†	(10.44)
Constant	45.90***	(4.75)	46.09***	(4.81)	46.58***	(4.79)	45.22***	(4.98)	45.73***	(4.97)
Chi square	603.04***		615.11***		713.95***		636.99***		719.16***	

^*a*^*n* = 95, 754 observations; Industry dummies and year dummies included (but not shown for readability); robust standard errors in parentheses

***p < 0.001, **p < 0.01, *p < 0.05, †*p* < 0.1

**Fig. 1:**
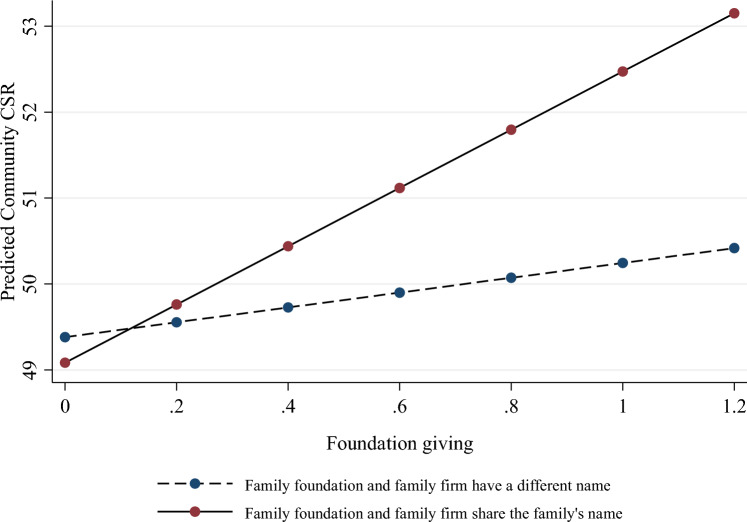
Interaction effect of foundation giving and name congruence

In H2 we predicted that the positive association between BOF’s family foundation giving and their family firm’s community CSR activity would become less positive for family firms that do not share their BOF’s name. The interaction effect in Table 2, Model 3 is positive (*β* = 2.53, *p* < 0.01), implying that the positive association between family foundation giving and community CSR activity is higher for family firms that share the BOF’s name and lower for those that do not. The effect remains consistent (*β* = 2.73, *p* < 0.01) when both interactions are entered in the full model (Model 5). For a better interpretation, we plot the interaction effect in Fig. 1, which shows firm’s community CSR activity on the y-axis and a related family foundation’s giving on the x-axis. The continuous and dashed lines represent family firms with and without name congruence, respectively. The figure shows that the positive association between family foundation giving and community CSR activity is less positive for family firms that do not have the same name as the BOF behind them (the average marginal effects calculation shows a slope of 0.84, *p* < 0.04) than for those that do share this name (slope of 3.32, *p* < 0.001). Regarding effect size, for a one-standard-deviation change from the mean in foundation giving, the difference in the expected mean score of community CSR is 1.69 points for firms named after the BOF behind them and 0.42 for those without such name congruence. All of this supports H2.

**Fig. 2:**
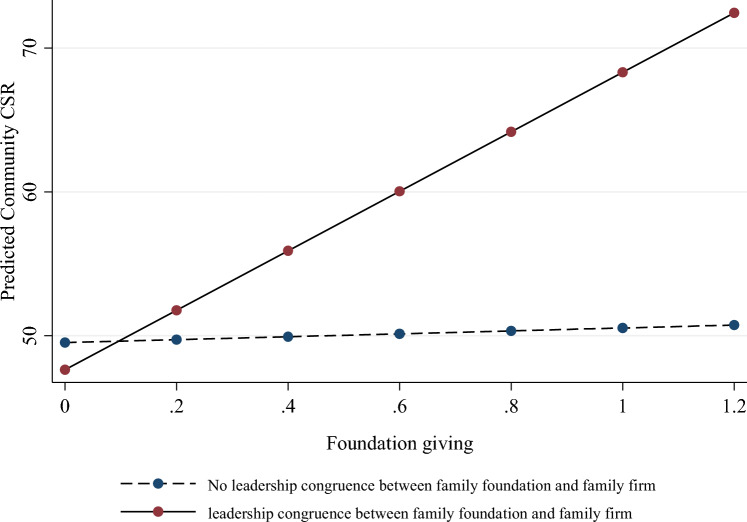
Interaction effect of foundation giving and leadership congruence

In H3, we predicted that the positive association between BOFs’ family foundation giving and their family firms’ community CSR activity would be more positive for family firms that shared leadership with their family foundation. In line with our hypothesis, the interaction effect in Model 4 was positive (*β* = 19.67, *p* < 0.06), and consistent in Model 5, where we included both interaction effects (*β* = 19.94, *p* < 0.06). We illustrate this interaction in Fig. 2. The figure shows that the association between family foundation giving and community CSR activity is more positive for firms that share leadership with their family foundation (the average marginal effects calculation shows a slope of 20.87, *p* < 0.048) than for firms that do not (slope 0.98, *p* < 0.043). Specifically, for a one-standard-deviation change from the mean in foundation giving, the difference in the expected mean score of community CSR activity is 10.44 points for firms that share the same leadership as the foundation, and 0.49 for those that do not. This supports H3.

### Robustness Analyses

We ran a number of further analyses to test the robustness of our results. First, we considered possible endogeneity concerns. One source of potential endogeneity was simultaneous causality (Bascle, 2008). Our dependent variable is measured in *t* + *1*, which helps account for the temporal precedence of foundation giving to the observed CSR outcomes. Still, we reran our models in several ways to account for this potential issue (see Appendix 1). First, we examined further lags of the dependent variable and found consistent results for both interactions on community CSR activity in *t* + *2*. Additionally, we tested whether community CSR activity influenced family foundation giving in future years and found that it was never a significant predictor in these models. While we are still careful not to claim causality, collectively these tests raise our confidence in the postulated direction of the relationship.

Another possible source of endogeneity is omitted variable bias. As is common in management research (Hill et al., 2021), there may be some variables unavailable to us (e.g., related to BOFs’ values), and thus not captured by our model, that drive both family foundation giving and community CSR activity. While the instrumental variable (IV) approach is one common way to address omitted variable problems (Bascle, 2008; Wooldridge, 2010), management research scholars who adopt it are often criticized for their inability to find suitable instruments that are both theoretically valid and methodologically strong (Eckert & Hohberger, 2022). Therefore, we follow recommendations to employ an IV-free Gaussian copula approach (see reviews by Becker et al., 2022; Eckert & Hohberger, 2022).^6^ This approach has the advantage of avoiding theoretical specifications (required for the IV approach) (Hill et al., 2021), and of not requiring additional variables, because “it directly models the correlation between the potentially endogenous regressor and the error term using a Gaussian copula … [and] provides a relatively simple way of identifying and correcting endogeneity biases in regression models” (Becker et al., 2022, p. 47). However, it can only be applied when: (1) the endogenous variable is *not* normally distributed, and (2) the regression residual of the estimation without the copula *is* normally distributed (Becker et al., 2022; Eckert & Hohberger, 2022). In our study, both requirements are satisfied. First, family foundation giving is highly skewed (skewness is 6.08), suggesting sufficient power to identify endogeneity (Becker et al., 2022). Supporting this, a Shapiro–Wilk test rejected that the variable is normally distributed (*p* < 0.000). Second, our results show that we cannot reject the null hypothesis of normality for the regression residual in the estimation without copula (*χ*^2^ = 1.40, *p* < 0.49).

This method has two stages: In the first stage, scholars should calculate the inverse normal of the cumulative distribution of the endogenous variable (Becker et al., 2022)—here, family foundation giving. In the second stage, the obtained variable (acting as the Gaussian copula for the endogenous variable) must be included as a control in the regression—here, predicting community CSR activity. We report our results in Appendix 2. We see these results as consistent with our main results, reported in Table 2. Specifically, family foundation giving increases community CSR activity, but the copula term is not significant (*p* < 0.19).

Further, while we theorized about the heterogeneity between BOFs by investigating the role of name and leadership congruence, we acknowledge the increasing importance of differentiating family businesses from each other (Jaskiewicz & Dyer, 2017; Neubaum et al., 2019), and have examined how such possible heterogeneity might influence our proposed relationships. First, our methodology did not originally distinguish between family firms and lone-founder firms (Miller et al., 2007). Thus, we ran additional checks, where we (1) introduced an additional control variable for lone-founder firms, (2) reran our results for a sub-sample excluding lone-founder firms, and (3) interacted the new control for lone-founder firms with family foundation giving. In all cases, the results (reported in Appendix 3) remained consistent, and the interaction between family foundation giving and lone-founder firm dummy was not significant. We ran similar checks (on sub-sample and interaction effects) for two factors that could distinguish between family firms, which we originally included as control variables. Specifically, we reran our results (1) distinguishing between first-generation family firms and others, and (2) using a stricter criterion for qualifying as a family firm (10% family ownership vs. the 5% threshold used in our initial analyses). Neither variable was a significant moderator when interacted with family foundation giving (results available on request). When we used these as criteria to create sub-samples, our results also remained largely unchanged.

## Discussion

This study was inspired by previous speculations in the literature (e.g., Atkinson & Galaskiewicz, 1988; Block & Wagner, 2014) that BOFs may see community social engagement via family foundation giving as a more efficient means of increasing their SEW, and that their exercise of a selective preference for this means of pursuing SEW would result in lower levels of firm community CSR activity (Block & Wagner, 2014). As this speculative relationship has important potential implications for our understanding of the ethics of BOFs and their firms, we sought to empirically investigate if and how BOFs’ family foundation giving affects their family firms’ community CSR activity. In times when wealthy BOFs face increasing scrutiny for their social engagement efforts, the consistent treatment of community stakeholders across (private and business) domains posited by our theorizing and supported by our empirics has important implications for research on family business and business ethics.

On a broader level, our work speaks to previous studies that suggested firms can be selective in their social engagements, taking some good actions in (apparently) unrelated domains in an attempt to offset bad perceptions in others (Cuypers et al., 2016; Scheidler et al., 2019). These studies theorized on the firm level, and examined consistency in firm CSR activities across different stakeholder domains. Relatedly, in the context of family business literature, some scholars similarly observed that BOFs can act responsibly and irresponsibly at the same time when they attend to different stakeholders (Cruz et al., 2014). For example, BOFs can be responsive to external stakeholder claims in order to preserve family reputation (a key SEW dimension), yet neglect claims of internal stakeholders because of the family’s desire to maintain control of the firm (another key SEW dimension). Our study argues that in the case of BOFs, this noted selectivity in social engagements *across* stakeholders does not translate to their social engagement *within* the same (and prioritized) community stakeholder group, as previous speculations suggest (Block & Wagner, 2014).

By complementing SEW theorizing with cue consistency theorizing, we explain why BOFs, even if pursuing SEW in line with their self-serving interests, will not solely consider the effects of one community engagement form over another in isolation, but will instead holistically engage with community stakeholders across domains to maintain perception of authenticity and associated SEW rewards. As such, BOFs will not be blind (as prior studies seem to assume they would be) to the potential negative SEW consequences of stakeholders observing them acting differently in different domains and judging their actions as inauthentic (Vazquez, 2018). Our findings that BOFs differ in prioritizing consistency depending on the ease with which stakeholders can connect a BOF’s community social engagement across the private and business domains provide evidence of the boundary conditions to these authenticity efforts.

Our study thus both validates the pursuit of SEW as an overarching motivation for BOFs’ community social engagements, and enriches this approach through the consideration of cue consistency. Future research could probe how BOFs prioritize the consistency of their potential engagement with more diverse stakeholders than our context considers. We also invite more CSR research exploring how ensuring consistency across CSR activities matters when firms engageactions with the same stakeholder group.

Our study also has important potential practical implications for BOFs attempting to maximize SEW benefits. We highlight the importance of consistency in BOF’s differing forms of social engagement in order for their community stakeholders to view them as authentic endeavors. Notably, to the extent that key stakeholders observing inconsistency between BOFs’ community social engagement efforts in the private domain, via family foundation giving, versus the business domain, via family firm community CSR activity, can develop a negativity bias, they are likely to perceive these families’ social engagement activities as hypocritical. This negative evaluation can yield negative SEW consequences in turn. However, ensuring this consistency becomes less important when there is no name or leadership congruence between a BOF (and its family foundation) and the firm(s) it owns. Hence, BOFs could reduce the risks associated with this cross-domain inconsistency by taking steps such as choosing a foundation name that doesn’t use their family and/or firm’s names, instead perhaps favoring a name that expresses the foundation’s mission. However, this approach may sacrifice some of the family legacy-building potential of naming a foundation after a BOF overall or a specific, important family member (Lungeanu & Ward, 2012), so BOFs would need to evaluate relative risks and benefits. BOFs that engage in inconsistent cross-domain activities could also adjust the compositions of their family foundations’ boards to minimize overlap with their firms’ leadership.

Finally, our study is timely for BOFs navigating crisis contexts, which seem to increase observers’ scrutiny of family foundation giving, and of family firms’ social engagement activities. Throughout the COVID-19 crisis, the US media has occasionally celebrated some acts of generosity by family foundations (e.g., the W.K. Kellogg Foundation’s plans to substantially increase their giving by more than US$1.7 billion; Steward and Kulish (2020)), but has also more systematically criticized this sort of philanthropy (e.g., the *Washington Post’s* 2020 survey of the 50 richest Americans noted that their aggregated US$1 billion donations during the crisis may sound generous but actually amounted to less than 0.1% of their combined wealth (Roberts and Hobson (2020)). Observers have also paid ample attention to employment maintained or destroyed by family firms in the communities in which they are thoroughly embedded (Firfiray & Gómez-Mejía, 2021). Our findings suggest that BOFs may be able to minimize this sort of scrutiny by, especially in hard times of crisis, ensuring consistency in their family foundation giving and family firm community CSR activity.

### Limitations and Future Research

While our results suggest that SEW considerations lead BOFs to holistically approach their community stakeholders, we cannot discern whether their community social engagement is instrumental (purely a means of obtaining SEW gains) or normative (motivated by true concerns about their communities’ wider social welfare) in any one instance or in general. This raises the question of whether SEW considerations can drive both instrumental and normative commitments towards community stakeholders. We encourage scholars to complement our research with qualitative work that advances SEW theorizing on the role of normative motives in BOFs’ social actions and impacts.

As is common in management research, we cannot make causal inferences, despite our efforts to guard against reverse causality. Community stakeholders expect family firms to exhibit high levels of community CSR activity (Bingham et al., 2011), so it would be difficult to argue that high levels of family firm community CSR activity will elicit the same level of scrutiny as high levels of private family foundation giving, and thus fuel high levels of family foundation giving. However, we do not rule out the possibility that some unobserved family firm factors may also influence family foundation community social engagement. We invite future research to investigate this possibility.

Relatedly, low levels of family foundation giving and high levels of family firm community CSR activity in principle also represent an inconsistency in community social engagement. Although in our context this specific inconsistency is less likely to trigger scrutiny than others, given that a low level of family foundation is less likely to draw criticism for its tax evasion potential, future research could investigate whether there are contexts in which such an inconsistency is also consequential for stakeholders’ evaluations of the authenticity of BOFs’ community social engagement.

While our theorizing assumes that the pursuit of SEW is the main driver of BOF community social engagement, we cannot ignore that different families may place varying levels of focus on SEW, or upon its distinct dimensions (Daspit et al., 2021; Gómez-Mejía & Herrero, 2022). The fact that our results remained unchanged after we included additional control variables (i.e., founder-led firms, family ownership, and family firm generation) that are traditionally used as indirect proxies for SEW (Gómez-Mejía et al., 2007) adds validity to our results. Nevertheless, previous studies have criticized the use of these variables to account for heterogeneity in BOFs’ pursuits of SEW (Debicki et al., 2016; Hauck et al., 2016; Miller et al., 2014). So we encourage future studies to use more direct proxies to explore how variations in, for instance, the importance BOFs give to ensuring that they maintain family control over their different entities, or their degree of identification with these entities influence the management of their community social engagement activities in the private and business domains.

Our focus on US family foundations renders the study context-dependent, as there is no globally applicable legal definition of such a foundation and results therefore may depend on national institutional frameworks (Anheier, 2018; Feliu & Botero, 2016; Rey-García & Puig-Raposo, 2013). Notably, in the US context family foundations are separate from firms the family owns, while in countries such as Germany foundations act as tools for transferring control of family firms across generations in a tax-efficient manner. Future research could explore the role of foundations in contexts in which they are less strictly separated from family firms than they are in the US (e.g., Germany), and how these contexts’ distinct environments might affect the existence of these foundations, their possible connection to family firms, and the actions they undertake.

We also encourage researchers to go beyond family foundations and investigate the relationship between community CSR activities and other potential vehicles for conducting family philanthropy in the private (e.g., individual or family donations) and/or the business domains (e.g., via corporate foundations or giving programs).

Finally, our sample consisted of large, listed US family firms and the elite class of wealthy BOFs behind them (Nason et al., 2019). This design allowed us to test arguments about the importance of BOFs’ visibility in the public eye and associated institutional pressures and public scrutiny. However, we recognize that using this sample limits the generalizability of our findings. Future research could investigate the need for consistency in community social engagement activities for less visible BOFs (e.g., those that own and operate smaller family firms, and/or those that own private businesses).

## Conclusion

This study complements the SEW approach with cue consistency considerations, all within the broader framework of institutional stakeholder theory, to theorize about the relationship between BOFs’ community social engagement in private (via their family foundations) and business domains (via family firm community CSR). We found that family foundation giving positively influences family firm community CSR activity, especially when there are strong visible associations between the BOF’s private- and business-domain vehicles for community social engagements (i.e., via congruence between the BOF’s name and that of its firm, and the BOF’s leadership in the family foundation and the firm).

This family-centered perspective (Jaskiewicz et al., 2017), which considers the social engagements of BOFs beyond their firms alone (Nason et al., 2019), is timely and appropriate because it redirects scholars’ prevailing attention on BOFs’ community social engagement through their family firms to the BOF’s private domain, revealing a more complete picture of BOFs’ social engagements (Van Gils et al., 2014). This in turn has important ethical implications as our study counters prior speculations regarding BOF's selectivity in socially engaging with their communities (Block & Wagner, 2014), showing instead their consistent and holistic approach to community social engagement across private and business domains.

## Appendix 1: Results for (1) Multiple lags of community CSR and (2) Reverse causality

**Table:**

Random-effects regression for community CSR (t + 2)		
Firm size (ln)	0.14	(0.51)
Return on assets	4.78	(3.96)
Tobin's Q	− 0.39	(0.45)
Debt-to-asset ratio	− 1.49	(2.57)
Firm age (ln)	1.12	(1.11)
Family ownership	0.93	(2.43)
Family management	− 0.88	(0.78)
Family board	− 3.05	(4.59)
Family generation	0.02	(1.06)
Corporate giving program	1.14	(1.25)
Corporate foundation	1.57	(1.57)
Foundation generosity	− 5.73*	(2.48)
Name congruence	0.45	(1.69)
Leadership congruence	− 1.25	(1.14)
Foundation giving	0.31	(0.67)
Found. giving x name congruence	2.45*	(1.22)
Found. giving x leadership congruence	14.40†	(8.14)
Constant	44.58***	(5.40)
Chi square	592.64***	

****p* < 0.001, ***p* < 0.01, **p* < 0.05, †*p* < 0.1

^a^*n* = 94, 681 observations; Industry dummies and year dummies included (but not shown for readability); robust standard errors in parentheses.

**Table:**

Random-effects regression for family foundation giving (t + 1)		
Community CSR	0.001	(0.00)
Firm size (ln)	− 0.03	(0.03)
Return on assets	0.10	(0.19)
Tobin's Q	0.06†	(0.03)
Debt-to-asset ratio	− 0.21	(0.14)
Firm age (ln)	0.06	(0.05)
Family ownership	− 0.17	(0.18)
Family management	0.05	(0.05)
Family board	− 0.42†	(0.24)
Family generation	− 0.03	(0.05)
Corporate giving program	0.04	(0.06)
Corporate foundation	− 0.06	(0.06)
Foundation generosity	0.21	(0.19)
Name congruence	− 0.03	(0.08)
Leadership congruence	− 0.02	(0.04)
Constant	0.16	(0.30)
Chi square	29.97	

****p* < 0.001, ***p* < 0.01, **p* < 0.05,†*p* < 0.1

^a^*n* = 93, 736 observations; Industry dummies and year dummies included (but not shown for readability); robust standard errors in parentheses

## Appendix 2: Instrument-free Gaussian copula estimates

**Table:**

	Model 2	
Firm size (ln)	− 0.38	(0.48)
Return on assets	7.35†	(3.79)
Tobin's Q	0.11	(0.40)
Debt-to-asset ratio	0.95	(2.25)
Firm age (ln)	1.18	(0.94)
Family ownership	− 1.93	(2.46)
Family management	0.33	(1.05)
Family board	0.63	(5.87)
Family generation	1.41	(1.39)
Corporate giving program	1.89†	(1.13)
Corporate foundation	3.11*	(1.36)
Foundation generosity	− 1.29	(3.13)
Name congruence	0.03	(1.46)
Leadership congruence	− 0.35	(1.53)
Foundation giving	2.54***	(0.65)
*Gaussian copula correction term*	− 0.68	(0.52)
Constant	46.51***	(4.74)
Chi square	635.95***	

****p* < 0.001, ***p* < 0.01, **p* < 0.05, †*p* < 0.1

^a^*n* = 95, 753 observations; Industry dummies and year dummies included (but not shown for readability); robust standard errors in parentheses

## Appendix 3: Robustness tests distinguishing lone-founder firms

**Table:**

	Sub-sample without lone-founder firms (*n* = 79, 614 obs)			Full sample + control for lone-founder firm (*n* = 95, 754 obs)			Full sample + control for lone-founder firm (*n* = 95, 754 obs)		
Firm size (ln)	0.04		(0.57)	− 0.17		(0.50)	− 0.29		(0.48)
Return on assets	1.49		(4.63)	6.75†		(3.76)	7.40†		(3.84)
Tobin's Q	0.76†		(0.42)	0.12		(0.39)	0.16		(0.40)
Debt-to-asset ratio	1.34		(2.42)	1.02		(2.21)	0.91		(2.28)
Firm age (ln)	1.44		(0.98)	1.23		(1.00)	1.19		(0.97)
Family ownership	− 0.77		(2.76)	− 2.20		(2.58)	− 2.25		(2.54)
Family management	− 0.18		(1.47)	0.48		(1.10)	0.44		(1.11)
Family board	1.69		(6.11)	0.21		(5.70)	0.12		(5.70)
Family generation	0.54		(1.55)	0.85		(1.43)	1.18		(1.42)
Corporate giving Program	1.82		(1.28)	1.99†		(1.17)	3.28*		(1.39)
Corporate foundation	3.17*		(1.37)	3.26*		(1.40)	1.92†		(1.15)
Foundation generosity	− 4.03		(3.14)	− 2.53		(3.34)	− 1.20		(3.19)
Name congruence	0.14		(1.49)	− 0.52		(1.44)	0.07		(1.45)
Leadership congruence	− 1.11		(1.65)	− 1.95		(1.40)	− 0.37		(1.52)
Lone-founder firm (dummy)				− 0.76		(1.25)	− 0.60		(1.37)
Foundation giving	0.23		(0.65)	0.66†		(0.40)	1.15†		(0.69)
Found. giving x name congruence	2.91*		(1.18)	2.73**		(0.96)			
Found. giving x leadership congruence	23.54		(14.83)	20.06†		(10.49)			
Found. giving x lone-founder firm							− 0.08		(0.68)
Constant	41.27***		(5.16)	46.38***		(5.14)	46.61***		(4.98)
Chi square	540.53***			751.19***			685.18***		

****p* < 0.001, ***p* < 0.01, **p* < 0.05, †*p* < 0.1

^a^Industry dummies and year dummies included (but not shown for readability); robust standard errors in parentheses

## Footnotes

1 This is evidenced by initiatives such as Forbes’s Philanthropy Score that seek to make billionaires’ generosity more transparent to the public (Çam 2018). Forbes 400 members are ranked not just on their total wealth, but also on their “generosity,” i.e., their giving as a percentage of net worth (Çam 2018).

2 This resonates with the profile of families in our sample. For example, in 2013, 73 of 97 family owners in our sample appeared on one of Forbes’s “richest” lists; of these, 61 were billionaires.

3 In our sample, 93 of 95 family foundations—but only a quarter of family firms—carried the BOF’s name.

4 In our sample, this corresponded to 30% of CEOs and 55% of board chairs, with 27% holding both positions, shared across family foundations and firms.

5 Our sample period begins when CSRHub began collecting data on firms’ CSR efforts.

6 We thank our editor for suggesting the Gaussian copula approach as the appropriate means of addressing endogeneity in our study.
